# Sensor Systems for Prognostics and Health Management

**DOI:** 10.3390/s100605774

**Published:** 2010-06-08

**Authors:** Shunfeng Cheng, Michael H. Azarian, Michael G. Pecht

**Affiliations:** 1 Center for Advanced Life Cycle Engineering (CALCE), University of Maryland, College Park, MD 20742, USA; E-Mails: chengsf@calce.umd.edu (S.F.C.); mazarian@calce.umd.edu (M.H.A.); 2 Prognostics and Health Management Center, City University of Hong Kong, Hong Kong; E-Mail: mgpecht@cityu.edu.hk

**Keywords:** sensor system, failure modes, mechanisms and effects analysis (FMMEA), Prognostics and health management (PHM)

## Abstract

Prognostics and health management (PHM) is an enabling discipline consisting of technologies and methods to assess the reliability of a product in its actual life cycle conditions to determine the advent of failure and mitigate system risk. Sensor systems are needed for PHM to monitor environmental, operational, and performance-related characteristics. The gathered data can be analyzed to assess product health and predict remaining life. In this paper, the considerations for sensor system selection for PHM applications, including the parameters to be measured, the performance needs, the electrical and physical attributes, reliability, and cost of the sensor system, are discussed. The state-of-the-art sensor systems for PHM and the emerging trends in technologies of sensor systems for PHM are presented.

## 1. Introduction

Prognostics and health management (PHM) generally combines sensing and interpretation of environmental, operational, and performance-related parameters to assess the health of a product and predict remaining useful life. Assessing the health of a product provides information that can be used to meet several critical goals: (1) providing advance warning of failures; (2) minimizing unscheduled maintenance, extending maintenance cycles, and maintaining effectiveness through timely repair actions; (3) reducing the life-cycle cost of equipment by decreasing inspection costs, downtime, and inventory; and (4) improving qualification and assisting in the design and logistical support of fielded and future systems [1].

The importance of PHM has been explicitly stated in the U.S. Department of Defense 5000.2 policy document on defense acquisition, which states that “program managers shall optimize operational readiness through affordable, integrated, embedded diagnostics and prognostics, embedded training and testing, serialized item management, automatic identification technology, and iterative technology refreshment” [2]. Thus, a prognostics capability has become a requirement for any system sold to the Department of Defense.

Traditionally, prognostics have been implemented using either a data-driven approach or a model-based approach [1]. The data-driven approach uses statistical pattern recognition and machine learning to detect changes in parameter data, isolate faults, and estimate the remaining useful life (RUL) of a product [1–4]. Data-driven methods do not require product-specific knowledge of such things as material properties, constructions, and failure mechanisms. In data-driven approaches, *in-situ* monitoring of environmental and operational parameters of the product is carried out, and the complex relationships and trends available in the data can be captured by data-driven methods without the need for specific failure models. There are many data-driven approaches, such as neural networks (NNs), support vector machines (SVMs), decision tree classifiers, principle component analysis (PCA), particle filtering (PF), and fuzzy logic [1].

Model-based approaches are based on an understanding of the physical processes and interrelationships among the different components or subsystems of a product [5], including system modeling and physics-of-failure (PoF) modeling approaches. In system modeling approaches, mathematical functions or mappings, such as differential equations, are used to represent the product. Statistical estimation techniques based on residuals and parity relations are then used to detect, isolate, and predict degradation [5,6]. Model-based prognostic methods are being developed for digital electronics components and systems such as lithium ion batteries [7], microprocessors in avionics [8], global positioning systems [9], and switched mode power supplies [10].

PoF-based prognostic methods utilize knowledge of a product’s life cycle loading conditions, geometry, material properties, and failure mechanisms to estimate its RUL [11–14]. PoF methodology is based on the identification of potential failure mechanisms and failure sites of a product. A failure mechanism is described by the relationship between the *in situ* monitored stresses and variability at potential failure sites. PoF-based prognostics permit the assessment and prediction of a product’s reliability under its actual application conditions. It integrates *in situ* monitored data from sensor systems with models that enable identification of the deviation or degradation of a product from an expected normal condition and the prediction of the future state of reliability.

Parameter monitoring and the analysis of acquired data using prognostic models are fundamental steps for these PHM methods, while sensor systems are the essential devices used to monitor parameters for PHM. PHM relies highly on the sensor systems to obtain long-term accurate *in situ* information to provide anomaly detection, fault isolation, and rapid failure prediction.

**Figure 1.**
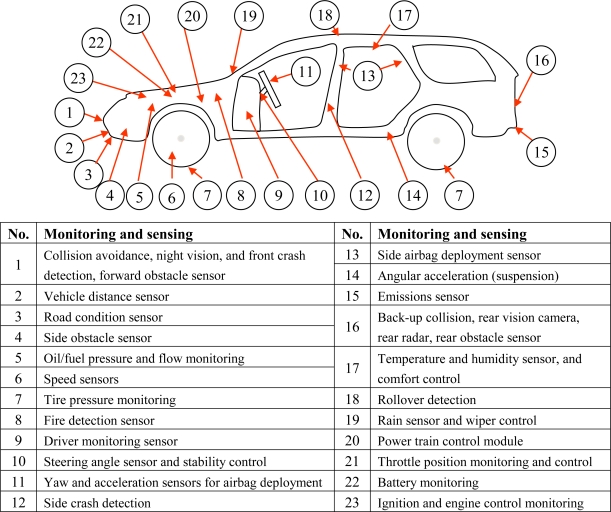
An example of PHM application for an automobile [15].

Firstly, PHM requires monitoring a large number of product parameters to evaluate the health of a product. Depending on the complexity of the monitored product, it is possible to monitor thousands of parameters in the entire life cycle of the product to provide the information required by PHM. These parameters include operational and environmental loads as well as the performance conditions of the product, for example, temperature, vibration, shock, pressure, acoustic levels, strain, stress, voltage, current, humidity levels, contaminant concentration, usage frequency, usage severity, usage time, power, and heat dissipation. In each case, a variety of monitoring features such as magnitude, variation, peak level, and rate of change may be required in order to obtain characteristics of parameters. Figure 1 is an example of PHM for an automobile to show the complexity of PHM application [15].

Secondly, parameter monitoring may be needed during all stages of the product life cycle, including manufacturing, shipment, storage, handling, and operation, since failures may occur due to the abnormal operational or environmental conditions of all these stages. Sensor systems provide the means by which the parameters can be monitored and the data can be acquired and processed.

Thirdly, PHM should have minimum adverse influences on the reliability of the monitored product and should have relatively low cost. This means that additional parts, such as sensor systems, should be selected carefully to minimize the adverse effects on the monitored host products. The features of PHM require many high performance sensor systems to continuously monitor record, analyze, and transfer a large amount of parameter data in the product’s life cycle.

In this paper, the considerations of a sensor system for PHM applications are discussed. Even though general considerations for sensor system selection can be applied to all branches of science and engineering, the features discussed above concerning PHM applications provide unique perspectives on these considerations and associated issues. The state-of-the-art of current commercially available sensor systems for PHM is presented by a survey. The emerging trends of the sensor systems for PHM are also predicted.

## 2. Considerations of Sensor System Selection for PHM

Considerations of sensor system selection for PHM may include the parameters to be measured, the performance needs of the sensor system, the electrical and physical attributes of the sensor system, reliability, and cost [16]. Sensor systems with multiple sensing abilities, miniature size and light weight, low power consumption, long range and high rate data transmission, large onboard memory, fast onboard data processing, low cost, and high reliability are specifically advantageous to PHM applications.

**Figure 2.**
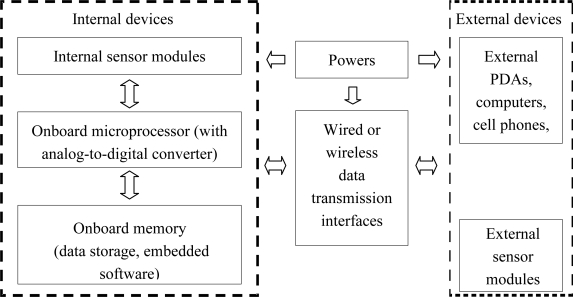
Integrated sensor system for PHM [17].

A generic sensor system will typically have sensing elements, onboard analog-to-digital converters, onboard memory, embedded computational capabilities, data transmission, and a power source or supply, as shown in Figure 2. In this figure, the internal sensor elements, onboard memory, and onboard processers are typical internal devices. The external memory, computers, and external sensor modules are typical external devices. The power sources can be internal, external, or a combination of both, and they provide power for the entire sensor system. The wired or wireless data transmission interfaces connect the external and internal devices. Not every PHM sensor system will necessarily contain all these elements, and not all sensor systems are suitable for the implementation of PHM. The user needs to understand the requirements of the PHM application to choose an appropriate sensor system.

### 2.1. Parameters to Be Monitored

In general, in order to assess the health of a product, the parameters to monitor for PHM include performance parameters (e.g., the speed of the fan in a laptop); physical characteristics (e.g., the pressure change in an oil pipeline or the strain of a printed circuit board when it is bended); electrical characteristics (e.g., the resistance of a resistor or the current through and voltage over a resistor); environmental conditions (e.g., temperature, vibration, pressure, acoustic levels, and humidity level); and operational conditions (e.g., usage frequency, usage severity, usage time, power, and heat dissipation). The parameters can also be classified based on different domains, as shown in Table 1.

For a specific PHM application, the parameters to be monitored can be identified based on their relationship to functions that are crucial for the safety of the product, are likely to be implicated in catastrophic failures, are essential for mission completeness, or result in long downtimes. Selection is also based on knowledge of the critical parameters established by past experience and field failure data from similar products and by qualification testing.

Systematic methods, such as failure modes, mechanisms, and effects analysis (FMMEA), can also be used to determine parameters that need to be monitored [18]. FMMEA is a methodology used to identify the critical failure mechanisms and models for all potential failure modes of a product under expected operational and environmental conditions. The output of the FMMEA process is a list of critical failure modes and mechanisms that enable us to identify the parameters to monitor and the relevant physics-of-failure models to predict the remaining life of the component.

**Figure 3.**
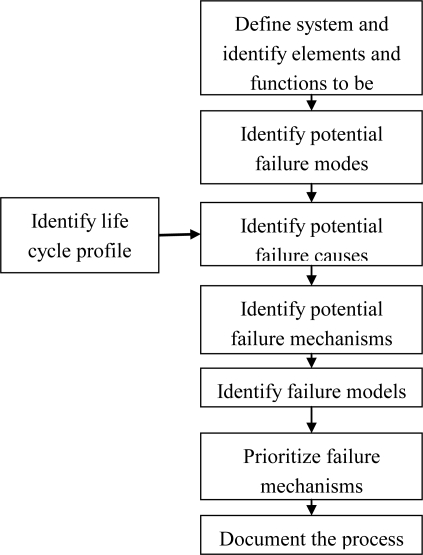
Flowchart of FMMEA process [18,19].

Figure 3 is a schematic flowchart of FMMEA. The product is divided into lower level subassemblies for investigation; these subassemblies are potential sites of failure. For each possible failure site, the functions and associated possible failure modes are analyzed. A failure mode is the way in which an item fails to perform its intended design function or performs the function but fails to meet its objectives [18–20]. Potential failure modes of an electronic product can be identified by analysis of the subassembly functions, and an understanding of how their impairment may be manifested. For example, for a cable in an electronic product, the failure modes may be stretching, breaking, kinking, or fraying.

For each failure mode, the potential failure causes should be identified. A failure cause is the specific process, design, and/or environmental condition that initiated the failure and whose removal will eliminate the failure. Possible failure causes are considered by investigation of the conditions of the life cycle of the product, including manufacturing/assembly, test, storage, transportation and handling, operation, and maintenance [18]. For example, the failure of multilayer ceramic capacitors (MLCCs), which are widely used in various devices, may be observed as parameter shifts, such as a decrease in insulation resistance or an increase in dissipation factor. Examples of the potential causes of these failures include improper ambient temperature and humidity conditions during storage or transportation. The improper bias voltage conditions in operation also contribute to failure.

Failure mechanisms are the processes by which specific combinations of physical, electrical, chemical, and mechanical stresses induce failures. The potential failure mechanisms are identified for each failure mode and site based on causes, loads, and design (geometry and material) [12]. Failure models are used as tools to assess failure propensity. In failure models, the stresses and the various stress parameters and their relationships to materials, geometry, and product life are considered. Each potential failure mechanism can be represented by one or more of failure models. FMMEA prioritizes the failure mechanisms based on their occurrence and severity in order to provide guidelines for determining the major operational stresses and environmental and operational parameters.

**Figure 4.**
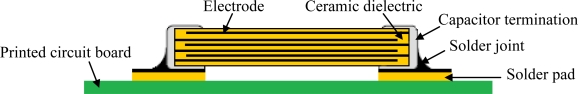
Schematic figure of PME-based MLCC [21].

The following is an example demonstrating the application of FMMEA to parameter identification for PHM. In this case, the parameters that can be used to monitor the health state of flexible-termination MLCCs under temperature-humidity-bias (THB) conditions are identified by FMMEA. Figure 4 is the schematic structure of the MLCC when it is soldered onto a printed circuit board [21]. The MLCC has precious metal electrodes (PME) made of silver-palladium. The ceramic dielectric material is between electrodes.

Table 2 is the FMMEA of the MLCC under THB conditions. Two failure mechanisms were dominant for PME-MLCCs under THB conditions. The first one was silver migration. Sato *et al.* [20] showed a three-step process for MLCC silver migration failure involving the formation of a microscopic crack, followed by penetration of moisture into the crack, and finally the movement of material (e.g., silver) that caused failure. Silver migration can cause a short, which would appear as a low insulation resistance value. The second failure mechanism was overall degradation of the dielectric of capacitors, which would also cause a lowering of insulation resistance and capacitance and an increase in the dissipation factor [22]. This degradation may be caused by moisture penetrating into voids and oxygen vacancies in the dielectric of the capacitors [22]. Based on this analysis, performance parameters (including insulation resistance, capacitance, and dissipation factor), environmental parameters (including temperature and humidity), and operational parameters (including bias voltage) should be monitored.

PHM requires integration of many different parameters to assess the health state, detect and isolate faults, and predict the remaining life of a product. If an individual sensor system can monitor multiple parameters, this would simplify PHM and reduce the cost of PHM. The sensing of multiple parameters refers to one sensor system that can measure multiple types of parameters such as temperature, humidity, vibration, and pressure. Structures that can conduct multiple sensing include the following: a sensor system that contains several different sensing elements internally; a sensor system with flexible, add-on external ports that support various sensor plug-in nodes; and combinations of these structures. For these structures, some common components can be shared such as the power supply, A/D converter, memory, and data transmission.

**Figure 5.**
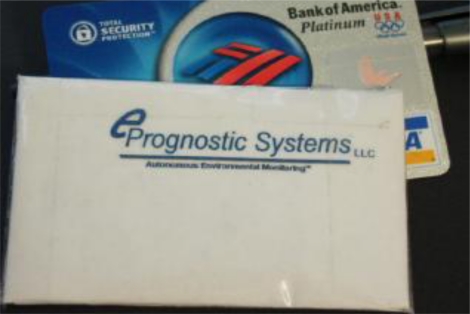
ePrognostic sensor system [17].

Figure 5 shows the ePrognostic sensor system, which includes multiple sensor elements. The ePrognostic sensor system can monitor multiple parameters used for prognostics, including temperature, humidity, motion, shock, and vibration. Table 3 shows the performance of this sensor system. In PHM applications, this sensor system can be used for the monitoring of the environmental and operational conditions of a product [17].

Fault detection and isolation is a necessary process in PHM to detect the occurrences of the faults and then identify the types, sites, and causes of the fault. Some faults and failure mechanisms can be identified by some sensor systems directly. For example, the resistance measurement can be used to isolate the open or short locations in a simple circuit, and corrosion sensors can use electrochemical impedance spectroscopy (EIS) to monitor the corrosion of structures directly [23]. For complex electrical packages, some techniques or sensor systems can be used to isolate the failure sites. For example, the Scanning Superconductive Quantum Interference Device (SQUID microscopy) can be used to detect shorts in the microprocessors; 3D x-ray radiography/tomography can image various levels of interconnections; and scanning acoustic microscopy can detect the interfacial delaminations and defects in the packages [24]. Other sensor systems using electromagnetic nondestructive testing technologies [25], ultrasonic guided wave technologies [26], or optical technologies [27] can detect cracks inside a product.

Fault isolation can also be done by using mathematical models, such as principle component analysis (PCA) [1,28] and residuals estimation [29], to analyze the data from “general” sensor systems. However, if the considered sensor system has the ability to detect and isolate the faults or failure mechanisms, it will improve the efficiency of PHM and provide more direct information. This type of sensor system may have a complete structure, including sensing elements, memory, processors, and display parts. They can monitor the corresponding parameters of the product, process the data using computers included in the system, display plots, and provide alarms. The selection considerations presented in this paper can also be used for the selection of this type of sensor systems.

### 2.2. Sensor System Performance

When the parameters to monitor are identified, the characteristics of these parameters, such as the possible range and frequency, should be understood. These characteristics can be obtained based on the historic records of the data or the specifications of the products. These features of the parameters should then be translated into the requirements for the performance attributes of sensor systems. Several relevant common performance attributes of sensor systems include the following:

- Measurement Range: the lowest and highest values of the measurands that the sensor can sense. The measurement range of the sensor systems should be wider than the actual range of the measurand.
- Dynamic Range: the ratio of the largest measurable output variation to the smallest distinguishable output variation, usually expressed in dB [30]; this is an important aspect of a sensor’s ability to respond to signals having both large and small amplitude variation.
- Accuracy: the closeness of agreement between the measurement and the true value of the measured quantity [31]. It can be presented as the error that is the difference between the measurement and the true value.
- Sensitivity: generally, the ratio between a small change in output to a small change in input, usually a unit change in input. Sensitivity represents the slope of the calibration curve [32]. In general, it can be described by the derivation of the output to the input. It may be a constant for all the inputs, but also may be different for different parts of the input.
- Repeatability: closeness of the agreement between the results of successive measurements of the same measurand carried out under the same conditions of measurement [31].
- Resolution: the minimal change of the input necessary to produce a detectable change in the output [32]. The resolution of the sensor is specified by the unit of the measured parameter or the percentage of the range of the measured parameter.
- Frequency Response: output-to-input ratio (the output power divided by the input power) of a sensor as a function of frequency and often given by dB [32]. It can be represented by gain-frequency response or phase-frequency response. The frequency response indicates the range of frequencies of the input for which the output is adequate (*i.e.*, does not decrease or increase the error due to the inability of the device to operate at a frequency or range of frequencies). The frequency range is between the lower and upper points where the amplitude of the signal has fallen off −3 dB, or 0.707 times of the input. The frequencies at these two points are cutoff frequencies. The frequency response range of sensor systems should be wider than the measured parameters.
- Hysteresis: the deviation of the sensor’s output at any given input point when the input is approached from two different directions [34].
- Linearity: the maximum deviation of the output functions from an ideal straight line [33].
- Response time: the time a sensor takes to react to a given input. This attribute represents how fast the sensor system can respond to the change of the measured parameter.
- Stabilization time: the time a sensor takes to reach a steady output upon exposure to a stable input.
- Sampling rate: the number of samples per second (or other unit) taken from a continuous signal to make a discrete signal.

PHM needs a high performance sensor system to identify the health of the monitored system and keep the uncertainty at a certain level. Gu *et al*. [35] studied the different sources of prognostic uncertainty and found that measurement inaccuracy by the sensor system was one of the main sources leading to uncertainty in PHM applications. Understanding the performance attributes of sensor systems mentioned above can help to determine the level of uncertainty caused by sensor systems and assist in controlling the overall uncertainty of the entire PHM application.

### 2.3. Physical Characteristics of Sensor Systems

The physical characteristics of a sensor system include its size, weight, shape, packaging, and mounting of sensors into the host. In some PHM applications, the size of the sensor may become one of the most significant selection criteria due to limitations of available space for attaching the sensor or due to the inaccessibility of locations to be sensed. In electronic products, due to the high-density components on the circuit board, it is difficult to mount large sensor systems to measure certain local parameters. In the motherboards of current computers, some sensors are embedded in the chips to save space and improve performance. For instance, the temperature of the CPUs and graphic processors are measured by thermal sensors built into the processor chips.

The weight of the sensor should also be considered in certain PHM applications, such as for vibration and shock measurements using accelerometers, since the added mass can change the system response. If a fixture is required to mount a sensor on a piece of equipment, the added mass of the sensor and fixture itself may change the system characteristics. When selecting a sensor system, users should determine the available size and weight capacity that can be handled by the host environment and then consider the size and weight of the entire sensor system, which includes the battery and other accessories such as antennas and cables.

**Figure 6.**
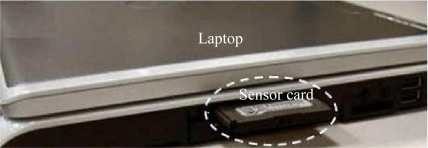
Example of sensing location [36] (Sensor card is put in the express card slot of a laptop).

Users should also consider the shape (round, rectangular, or flat) of the sensor system; the packaging materials, such as metal or plastic; and the method for attaching or mounting the sensor, for example, glue, adhesive tape, magnets, fixtures, or screws (bolts) to affix the sensor system to the host. The following is an example explaining these requirements for sensor systems in a PHM application. In this example, the PHM method is used to identify the health status of a laptop by monitoring the internal environmental parameters, such as temperature, shock, and humidity. The express card slot, as shown in Figure 6, on the side of the laptop is used to place the sensor system to measure these parameters. Based on this placement, the sensor system must be in the shape and size of the typical express card, should have a plastic or metal outside packaging, should be able to transmit the data through the card interface or wireless protocol, and should be able to be mounted in the slot.

### 2.4. Functional Attributes of Sensor Systems

The electrical attributes of the sensor systems that should be considered include the following: onboard power and power management ability; onboard memory and memory management ability; programmable sampling rate; the rate, distance, and security of data transmission of the sensor system; and onboard data processing capability. Each of these attributes will be discussed below.

#### 2.4.1. Power and Power Management

Power consumption is an essential characteristic of a sensor system as it determines how long the sensor system can function without connection to an external source of power. The power consumption of sensor systems can be divided into three main domains: sensing, communication, and data processing [37]. In these three domains, wireless data communication consumes the most energy. In order to attain the required duration of operation in such applications, a sensor system must have a sufficient power supply and the ability to manage power consumption.

Sensor systems can be divided into two main categories with respect to their power sources: non-battery-powered and battery-powered. Non-battery-powered sensor systems are typically either wired to an external AC power source or they use power from an integrated host system. For example, a temperature sensor is often integrated within the microprocessor on a motherboard and utilizes the computer’s power supply.

Battery-powered sensor systems are equipped with an onboard battery. Replaceable or rechargeable batteries allow sensor systems to operate continuously without replacing the entire system. Rechargeable lithium ion batteries are commonly used in battery-powered sensor systems. In some situations, the battery is inaccessible. The use of batteries with a larger capacity or standby batteries may be required in such applications.

Power management is used to optimize the power consumption of the sensor system in order to extend its operating time. Power consumption varies for different operational modes of the system (e.g., active mode, idle mode, and sleep mode). The sensor is in active mode when it is being used to monitor, record, transmit, or analyze data. The power consumed for sensing varies depending on the parameter sensing methods and sampling rate. Continuous sensing will consume more power, while periodic or event-triggered sensing will consume less power. A higher sampling rate will consume more power because it senses and records data more frequently. Additionally, wireless data transmission and onboard signal processing will consume more power.

In its idle state, a sensor system consumes much less power than during active mode. Sleep mode consumes the lowest power. The tasks of power management are to track and model incoming requests or signals in order to determine when to switch between the active state and idle state, how long the idle state will be maintained, when to switch to the sleep state, and when to wake up the system. For example, in continuous sensing, the sensing elements and memory are active, but if data transmission is not required, the sensor system can be put into sleep mode. Power management will wake up the data transmission circuit when it receives a request.

#### 2.4.2. Onboard Memory and Memory Management

Onboard memory is the memory contained within a sensor system. It can be used to store collected data as well as information pertaining to the sensor system (e.g., sensor identity, battery status), which enables the sensor system to be recognized and to communicate with other systems. Firmware (embedded algorithms) in the memory provides operating instructions to the microprocessor and enables it to process the data in real time. Onboard memory allows for much higher data sampling and save rates.

Memory requirements are affected by sensing mode and sampling rate. Sensor systems should allow the user to program the sampling rate and set the sensing mode (*i.e.*, continuous, triggered, and threshold). These settings affect the amount of data stored in the memory.

Memory management allows a user to configure, allocate, monitor, and optimize the utilization of memory. For multiple-sensing sensor systems, the data format will often depend on the sensing variable. Memory management should be able to distinguish various data formats and save them into corresponding areas of the memory. For example, the sampling rate, time stamp, and data range of temperature are different from those of vibration data. In the memory, these different data may be stored separately based on algorithms in order to make them easy to identify. Memory management also should have the ability to show the usage status of the memory, such as the availability percentage, and indicate when the memory is becoming full.

The sampling mode determines how the sensor monitors the parameters and at what times it will actively sample the measurand. Commonly used sampling modes include periodic and event-triggered sampling. The sampling rate defines the number of samples per second (or other unit of time) taken from a continuous signal to make a discrete signal. The combination of sampling mode and sampling rate controls the sampling of the signal.

Programmable sampling modes and rates are preferred for PHM applications, since these features affect diagnostic and prognostic power consumption and memory requirements directly. Under the same sampling mode, a low sampling rate consumes less power and memory than a high sampling rate. But a sampling rate less than twice the frequency of operation leads to distortion in reconstructed signals and may reduce the likelihood of capturing intermittent or transient events that are needed for fault detection. Additionally, if the user wants to utilize a sensor, for example, to monitor vibration and temperature at the same time, the sensor system should allow the user to set the sampling mode and rate for these different types of parameters individually.

#### 2.4.3. Onboard Data Processing

Signal processing consists of two parts: one is embedded processing that is integrated into the onboard processor to enable immediate and localized processing of the raw sensor data; the other is processing conducted in the host computer. When selecting sensor systems, one should consider both of these functions.

Onboard processing can significantly reduce the number of data points and thus free up memory for more data storage. This in turn reduces the volume of data that must be transmitted out to a base station or computer, and hence results in lower power consumption by data transmission. In the case of a large number of sensor systems working in a network, this would allow decentralization of computational power and facilitate efficient parallel processing of data.

Onboard processing can also facilitate efficient data analysis for environmental monitoring applications. Onboard processing can be set to provide real-time updates for taking immediate action such as powering off the equipment to avoid accidents or catastrophic failures. It can also be set to provide prognostic horizons to conduct future repair and maintenance activities.

Currently, onboard signal processing includes feature extraction (e.g., rainflow cycle counting algorithm), data compression, fault recognition, and fault prediction. Ideally, the onboard processor should display its calculation results and execute actions when a fault is detected, and it should be programmable.

However, the abilities of the onboard processor are limited by some physical constraints. One constraint is the available power. If processing requires extended calculation and high calculating speeds, it will consume much more power. The other constraint is onboard memory capacity. Running complex software requires a lot of memory. These two constraints make it challenging to embed complex algorithms into onboard processors. However, even using simple algorithms and routines to process raw sensor data can achieve significant gains for *in-situ* analysis.

#### 2.4.4. Data Transmission

Once data is collected by the sensor system, it is typically transmitted to a base station or computer for post-analysis. In general, the methods for data transmission are either wireless or wired. Wired data transmission can offer high speed transmission, but this transmission is limited by the need for transmission wires, and the cost is increased by wires. Wireless transmission has emerged as a promising technology that can impact PHM applications. Wireless transmission refers to the transmission of data over a distance without the use of a hard-wired connection. The distances involved may be short (a few meters, as in a television remote control) or very long (thousands or even millions of kilometers for radio communications). Wireless sensor systems can be used to remotely monitor inhospitable and toxic environments and transmit measured data to a centrally located processing station. Also, since wireless sensor systems are not dependent on extensive lengths of wires for the transmission of measurement data, they save installation and maintenance costs. The advantage of wireless sensor systems can be greatly enhanced by embedding micro-controllers that have data analysis capabilities within the wireless sensor.

A lot of wireless technologies can be used for wireless data transmission of sensor systems, for example, Radio Frequency Identification (RFID), Bluetooth, Wi-Fi (IEEE 802.11), Ultra-Wideband (UWB), Certified Wireless USB (WUSB), WiMax (Worldwide Interoperability for Microwave Access and IEEE 802.16), and Zigbee (IEEE 802.15.4). When selecting the wireless technology to use for a particular application, the user should consider the range and rate of communication, power consumption, ease of implementation, and data security.

An RFID sensor system combines the RFID tag with the sensing element. An RFID tag is an object that can be attached to or incorporated into a product, animal, or person for the purpose of identification or monitoring using radio waves [38]. This RFID tag uses sensing elements to detect and record temperature, humidity, movement, or even radiation data, and then utilizes RFID to identify the sensor as well as to transfer the raw data or processed data. RFID systems use many different frequencies, for example, low-frequency (around 125 KHz), high-frequency (13.56 MHz), and ultra-high-frequency (UHF, higher than 860 MHz). In order to communicate to readers, RFID tags have to be tuned to a frequency in common with the readers. [39].

The ePrognostic sensor system shown in Figure 5 is an RFID-based sensor system. The multiple sensing elements, such as temperature, vibration and humidity, are embedded in the RFID tag. Monitored data is transferred by RFID to a reader connected to a computer, The RF frequency of an ePrognostic sensor tag is typically 915 MHz or 2.4 GHz. The communications range from a few feet (for security) to 100 m.

Bluetooth is an RF-based wireless data transmission technology. It operates in the 2.4 GHz to 2.485 GHz ISM (industrial, scientific, medical) band utilizing low-transmit power radios and the frequency-hopping spread spectrum technique [40]. Bluetooth devices hop though 1,600 frequency channels per second, of which 800 channels are transmit channels and the other 800 channels are receive channels. The channels span 79 MHz with 1 MHz spacing between the neighboring channels. Thus, Bluetooth is designed to be functional even in very noisy RF environments [41]. The data rate is 1 Mbps for Version 1.2, up to 3 Mbps supported for Version 2.0 + Enhanced Data Rate (EDR), and up to 24 Mbps supported for Version 3.0 + Higher Speed (HS) [41].

Wi-Fi (IEEE 802.11) is another RF-based wireless technology that can be used in sensor systems. A typical Wi-Fi setup includes one or more access points (APs) and one or more clients (computers, video game consoles, mobiles, sensors, *etc*.). The primary job of an access point is to broadcast a wireless signal that computers can detect and “tune into”. Wi-Fi networks operate in the 2.4 GHz (802.11b/g/n), 5 GHz (802.11a/n), and 3.7 GHz (802.11.y) radio bands, with 11 Mbit/s (802.11b), 54 Mbit/s (802.11a/g/y), or up to 600 Mbit/s (802.11n) data rates, respectively. The outside transfer range can be up to 120 m (802.11.a), 140 m (802.11.b/g), and 250 m (802.11.n) [42].

Zigbee (IEEE 802.15.4, Low-rate Wireless Personal Area Network (WPAN) standard) is used for industrial controls (for example, process control and energy management), embedded sensing, medical data collection, smoke and intruder warning, building automation (for example, access control and energy monitoring), and home automation (e.g., smart lighting, temperature control, and safety and access control). Zigbee works at 2.4 GHz and 868/915 MHz. The data rate is 250 Kbps at 2.4 GHz, 40 Kbps at 915 MHz, and 20 Kbps at 868 MHz. The transmission range is between 10 and 75 m and up to 1,500 m for Zigbee Pro, although it is heavily dependent on the particular environment [43].

An Ultra-Wideband (UWB) transmitter works by sending billions of pulses across a very wide spectrum of frequencies several GHz in bandwidth. The corresponding receiver then translates the pulses into data by listening for a familiar pulse sequence sent by the transmitter. UWB’s combination of a larger spectrum, lower power, and pulsed data improves speed and reduces interference with other wireless spectra. In the United States, the Federal Communications Commission (FCC) has mandated that UWB radio transmissions can legally operate in the range from 3.1 GHz up to 10.6 GHz at a limited transmit power of −41 dBm/MHz [44].

Certified Wireless USB is the specification of a wireless extension of the USB standard intended to further increase the availability of general USB-based solutions. It enables products such as personal computers, consumer electronics, and mobile devices to connect using a common interface at up to 480 Mbit/s at 3 m and 110 Mbit/s at 10 m. At close range, it is the same rate as Hi-Speed USB. Wireless USB was designed to operate in the 3.1 GHz–10.6 GHz range. Certified wireless USB has power management strategies. Sleep, listen, wake, and conserve modes ensure that devices use only the minimum power necessary. All Certified Wireless USB products are required to encrypt their data transmissions [45].

The security of wireless data transmission is an important factor that should be considered. The standard approach is to encrypt the data transmitted by the sensors. In addition, it might also be necessary to provide authentication mechanisms in order to guarantee that the data source is the claimed sender [46,47]. Walters *et al.* [48] classified different security risks of wireless sensor networks and described defensive methods to protect data transmission. One should evaluate the security strategy of the wireless sensor system or customize the security level to protect data during transmission.

Currently, hybrid data transmission combines wired data transmission and wireless data transmission. This arrangement can represent a compromise that improves data transmission, power requirements, and cost. Wired transmission offers high speed transmission and consumes low energy. Wireless data transmission can offer convenient data communication and eliminate the need for wire, but it consumes more power. There are trade-offs to be made for any given application. For example, many sensor systems transfer data from a sensor to a receiving device wirelessly, and then the receiving device transfers the data to a computer by a wired USB port.

### 2.5. Cost

The selection of the proper sensor system for a given PHM application must include an evaluation of the cost. The cost evaluation should address the total cost of ownership including the purchase, installation, maintenance, and replacement of sensor systems. In fact, the initial purchase cost of a product can be less than 20% of the product’s total lifetime cost [49].

In addition to cost of ownership, justifying the cost of sensors can be quite challenging for products produced in large volumes. Prognostics and health management (PHM) methods for these products are more complex because more parameters need to be monitored and more sensor elements or systems are required. In this case, the total cost could differ considerably for different sensor system integration strategies. One strategy is that the user purchases individual sensor systems at a low cost and then integrates them. A second strategy is to purchase integrated sensor systems with multiple sensing abilities, onboard power, memory, processing capabilities, and wireless data transmission. Typically, the cost for integrated sensor systems will be lower than the total cost of individual sensor systems and the integration of these individual sensor systems. The third strategy is to integrate or build the sensor systems in the product as part of the product. This provides the lowest cost for sensor systems, but it will increase the cost for the product due to new hardware, software, and qualification procedures, *etc*.

### 2.6. Reliability

Failed sensor systems provide incorrect or incomplete data and cause PHM to generate wrong detections, alarms, and predictions. If the sensor system is used to measure a critical parameter or is installed at a limited access location, the adverse impacts to PHM will be more severe. The reliability of a sensor system requires the ability of the sensor system to perform a necessary function under stated conditions for a stated period. However, reliability information, such as the mean time between failures (MTBF) and failure rate under certain environmental and operational conditions, is rarely specified by the sensor system manufacturers.

One strategy to improve the reliability of sensor systems is to use multiple sensors (redundancy) to monitor the same product or system. By using redundancies, the risk of losing data due to sensor system failure is reduced, but the cost increases.

Some technologies, such as sensor validation, can also improve the reliability of sensor systems. For example, sensor validation [50] is used to assess the integrity of a sensor system and adjust or correct it as necessary. This functionality checks the sensor performance and ensures that the sensor system is working correctly by detecting and eliminating the influence of systematic errors. When selecting a sensor system, the user should check to see if the sensor system has the validation functions.

While it is essential to consider the reliability of sensor systems, it is equally necessary to consider the effects of the sensor system on the reliability of the product it is intended to monitor. Sensor systems that are heavy may reduce the reliability of the circuit boards they are attached to over time. In addition, the method of attachment (soldering, glue, or screws) can reduce the reliability of the product if the attachment material is incompatible with the product’s construction materials.

## 3. State-of-the-Art Sensor Systems for PHM Implementation

In 2008, a survey was conducted by the authors to determine the commercial availability of sensor systems that can be used in PHM for electronic products and systems. The survey only included commercially available sensor systems having features desirable for PHM.

The survey results showed the characteristics of 33 sensor systems from 23 manufacturers. The sensor system characteristics included sensing parameters, power supply and power management capability, sample rate, onboard memory, data transmission method, availability of embedded signal processing software, size, weight, and cost. The data for each sensor system was collected from the manufacturer’s website, product datasheets, e-mails, and evaluations of demo products. The data is listed in Appendix A of [1].

Key findings from the survey are that state-of-the-art sensor systems: (1) can autonomously perform multiple functions using their own power management, data storage, signal processing, and wireless data transmission; (2) have multiple, flexible, or add-on sensor ports that support various sensor nodes to monitor various parameters such as temperature, humidity, vibration, and pressure; (3) have onboard power supplies, such as rechargeable or replaceable batteries; (4) have onboard power management, allowing control of operation modes (active, idle, and sleep), and programmable sampling modes (continuous, triggered, or threshold) and sampling rates; (5) have diverse onboard data storage capacity (flash memory), from several KB to more than hundreds of MB; (6) have embedded signal processing algorithms that enable data compression or simplification prior to data transfer; and (7) use various wireless technologies including RFID, Bluetooth, Wi-Fi, Zigbee, Ultra-Wideband, and Wireless USB.

The survey also found that current sensor systems should be improved in several aspects for PHM applications. The first areas are size and weight. PHM for an electronics system with a high density of components requires small-size and light-weight sensor systems that can be placed on the circuit board and that minimize the weight increase and potential adverse effects on the reliability of the monitored electrical system.

Onboard power is also one of the main limitations of current commercially available sensor systems, especially for wireless data transmission sensor systems. The main onboard power is a battery, which needs be replaced or recharged when it is used up. Higher capacity batteries with small size and light weight or battery-free power are needed for sensor systems to operate longer in PHM applications.

Onboard data processing ability should also be improved. In the survey, only a few sensor systems had onboard data processing ability, and these only had very simple functions, for example, data reduction. Onboard data processing provides timely information about the health of the system and can reduce the cost of the entire PHM application. Onboard data processing requires high-speed processors, large-capacity memory, more power, and processing algorithms. The processing algorithms can be developed based on specific PHM applications.

## 4. Emerging Trends in Sensor Technology for PHM

In general, PHM application requires that sensor technology should be headed toward extreme miniaturization, battery-free power or ultra-low power consumption, and intelligent wireless networks. Since electronic components and systems continue to decrease in size, sensors to monitor their environments and operation will also become smaller and weigh less in order to be integrated. As Micro Electro Mechanical Systems (MEMS) or Nano Electro Mechanical Systems (NEMS) and smart material technologies mature, MEMS sensors or nanosensors will integrate the sensing element, amplification, analog-to-digital converter, and memory cells into one microchip. The fabrication of MEMS and NEMS will offer significant advantages in terms of integration with electronics, fabrication of arrays of sensors, small size of individual devices, low-power consumption, and lower costs [51].

With the development of new materials and energy technologies, battery-free sensor systems are being considered, especially for use in embedded, remote, and other inaccessible monitoring conditions. Battery-free sensor systems will be developed based on ultra-low power electronics and energy-harvesting technologies.

Ultra-low power electronics will enable future sensor systems to consume much less power. A lot of new ultra-low power consumption technologies are emerging. For example, in June 2009, STMicroelectronics announced a new ultra-low power technology platform for building a range of 8-bit and 32-bit microcontrollers, which will enable future generations of electronic products to consume less power, meet evolving energy-efficiency standards, and operate for longer times from their batteries. This new platform is built on a 130nm process, which ST has further optimized with ultra-low leakage transistors for logic functions, low voltage transistors for analog functions, innovative low power embedded memory, new low-voltage low-power standard peripherals, and an innovative power management architecture. “These enhancements dramatically reduce dynamic and static power consumption, enabling forthcoming families of microcontrollers delivering better performance per Watt than the most frugal low-power devices on the market today” [52]. The emerging ultra-low power technologies enable the production of ultra-low power consumption chips for all kinds of electronic products.

Energy harvesting is a process to extract energy from the environment or from a surrounding system and convert it to usable electrical energy. Current energy harvesting sources include sunlight, thermal gradient, human motion, body heat, wind, vibration, radio power, and magnetic coupling. Several excellent articles reviewing possible energy sources for energy harvesting can be found in the literature [53–57]. Some large-scale energy harvesting schemes such as wind turbines and solar cells have made the transition from research to commercial products. The interest in small-scale energy harvesting for embedded sensor systems, such as implanted medical sensors and sensors on aerospace structures, is increasing.

Basic effects used in energy harvesting include electromagnetic, piezoelectric, electrostatic, and thermoelectric effects. For example, the mechanical vibration inside a device or ambient mechanical vibration can be converted into electric energy by piezoelectric materials or electromagnetic induction.

Piezoelectric materials form transducers that are able to interchange electrical energy and mechanical vibration or force. The electromagnetic induction systems are composed of a coil and a permanent magnet attached to a spring. The mechanical movement of the magnet, which is caused by a device or ambient vibration, induces a voltage at the coil terminal, and this energy can be delivered to an electrical load. The thermal energy is often converted into electric energy by thermoelectric generators (TEGs). With recent advances made in nanotechnologies, the fabrication of MEMS-scale TEG devices has been actively studied. The combined use of several energy harvesting sources in the same device can increase the harvesting capabilities in different situations and applications and can minimize the gap between the required and harvested energy [58].

Distributed sensor networks (DSNs) consist of multiple sensor nodes that are capable of communicating with each other and collaborating on the same sensing goal [59]. The advantage of a DSN is that it allows data from multiple sensors to be combined or fused to obtain inferences that may not be possible from a single sensor. The sensor nodes in a DSN are organized into a cooperative system. The nodes can communicate with each other and have the ability to self-organize.

Wireless transmissions, such as Wireless USB, are being transferred to sensor products. The development of wireless transmission technology will produce intelligent wireless sensor networks with characteristics of long distance, high transmission rate, ultra-low power consumption, more secure data communication, and high-speed data fusion processing. Furthermore, future smart sensor nodes will be highly intelligent, with more functions than today’s sensors [60]. They will have built-in diagnostic and prognostic capabilities, which will make the entire sensor network more functional. The integration of all of the above technology accelerates the development of wireless intelligent sensor networks [61].

## 5. Discussion

This paper mainly focuses on commercially available sensor systems that can be used for PHM applications with minor or no design modifications. There are several limitations with these add-on sensor systems. For example, they cannot be effectively integrated with the monitored product and hence cannot sense critical variables that are integral to product operation and performance, such as voltage rails, power buses, *etc*. One way for PHM application to overcome this limitation is to identify the correlations between the parameters that can be monitored by add-on sensor systems and the parameters integral to product operation and performance based on historic data or training data. Based on the correlation, PHM can infer changes in critical parameters based on the parameters monitored by add-on sensor systems. For example, Kwon *et al*. [62] demonstrated that monitored RF impedance increased as a physical crack propagated across the solder joint under stress conditions, and characterized the extent of physical solder joint degradation associated with the changes in RF impedance. The other method is for PHM to integrate the information offered by the add-on sensor systems and the information obtained from the system buses of the product. For example, when applying PHM for a laptop, a sensor system that can sense temperature, humidity, shock, and position can be integrated into a card that can be inserted into the card slot of the laptop to monitor these parameters. On the other hand, PHM also obtains the information acquired by BIOS from the system bus, for example, the internal temperature of the CPU and GPU, fan speed, memory usage condition, CPU usage condition, *etc*.

Other limitations, such as accessibility to the best sensing locations due to limited space for sensing or inhospitable or toxic environments, can be overcome with the development of sensor technologies that can make smaller and more powerful wireless sensor systems.

The selection of a sensor system for PHM applications requires analysis of the application, identification of the parameters to be monitored and all the requirements for the sensor systems, and prioritization of these requirements based on the specific application. Then, sensor system candidates should be identified and evaluated based on these requirements. Finally, some trade-offs must be made to select proper sensor systems.

## 6. Conclusions

PHM is an enabling discipline that assesses the reliability of a product in its actual life cycle. PHM for a product is a long-term effort that requires monitoring the product by continuous or periodic measuring, sensing, recording, and analyzing of different parameters in its life cycle to assess the health status of the product, identify abnormal conditions, and predict the remaining life of the product. In PHM applications, sensor systems are essential devices for conducting *in situ* monitoring of the actual life cycle of a product. PHM requires that sensor systems be highly integrated with multiple sensing abilities, low power consumption, low cost, long-range and high-rate data transmission (wireless or wired), large onboard memory capacity, fast onboard data processing abilities, miniature weight and size, and high reliability.

A survey of the current commercially available sensor systems identified the state of the art of sensor systems. Many sensor systems have multiple functions, such as multiple sensing capabilities, onboard power and power management ability, onboard memory and management ability, wireless data transmission, and onboard data processing ability. However, the survey also identified several unmet needs of sensor systems for PHM applications, including size and weight of the sensor systems and the limitations of onboard power supply, memory capacity, onboard data processing ability, *etc*. Several technologies such as MEMS or NEMS, energy harvest techniques, ultra-low power consumption circuits, and intelligent wireless sensor network techniques, have been emerging to provide more powerful sensor systems in the future. The trend in sensor systems is toward extreme miniaturization, wireless transmission with long range and high rate, low cost, ultra-low power consumption, battery-free power supply, stronger onboard data processing ability, more intelligence, and networking.

In a specific PHM application, the user can identify the parameters to be monitored by a failure mechanism-based method called failure modes, mechanisms, and effects analysis (FMMEA). The requirements of PHM applications for sensor systems regarding sensor performance, electrical and physical attributes, reliability, cost, and availability of sensor systems must also be understood. Some trade-offs must be made to select proper sensor systems as well.

## Figures and Tables

**Table 1.** Examples of parameters for PHM applications [17].

**Domain**	**Examples**
Mechanical	Length, area, volume, velocity or acceleration, mass flow, force, torque, stress, shock, vibration, strain, density, stiffness, strength, angular, direction, pressure, acoustic intensity or power, acoustic spectral distribution
Electrical	Voltage, current, resistance, inductance, capacitance, dielectric constant, charge, polarization, electric field, frequency, power, noise level, impedance
Thermal	Temperature (ranges, cycles, gradients, ramp rates), heat flux, heat dissipation
Chemical	Chemical, species concentration, gradient, reactivity, mess, molecular weight
Humidity	Relative humidity, absolute humidity
Biological	pH, concentration of biological molecules, microorganisms
Electromagnetic radiation and ionizing radiation	Intensity, phase, wavelength (frequency), polarization, reflectance, transmittance, refractive index, distance, exposure dose, dose rate
Magnetic	Magnetic field, flux density, permeability, direction, distance, position, flow

**Table 2.** FMMEA of the MLCC under THB condition.

**Potential Failure Sites**	**Potential Failure Modes**	**Potential Failure Causes**	**Potential Failure Mechanisms**
Electrodes	Short, decrease in resistance, decrease in capacitance, crack	Thermal stress, moisture, bias voltage, bending of printed circuit board	Silver migration, corrosion, fatigue
Ceramic dielectric	Decrease in insulation resistance, decrease in capacitance, increase in dissipation, crack		Aging of ceramic dielectric

**Table 3.** Performance of the ePrognostic sensor tag [17].

**Measured Parameters**	**Performance**
Temperature	Range: −10 °C to 60 °C (standard); 10 °C to 100 °C for special order option; Accuracy: ±1 °C over the full standard range; Programmable sample time intervals: from 10 s to 24 h
Motion	3D motion sensing; Sensitivity is multiple step g-force level from 1.5 g to 10 g; g-force, motion, and time stamp are recorded
Shock	10 G maximum measurement; 3D sensing; Preprogrammed sensitivity: up to 10 g
Higher-level Shock	200 G shock can be measured in single dimension; Preprogrammed sensitivity: up to 200 g
Vibration	Maximum frequency approaches 2kHz, with an accuracy of ±5% at top range
Relative Humidity	Range: from 10%RH to 90%RH;Programmable sample time intervals: from 10 s to 24 h; Accuracy: ±10%RH over the full temperature range (−10 °C to 100 °C)
